# *In vivo* antimalarial activity of ethanolic leaf extract of *Stachytarpheta cayennensis*

**DOI:** 10.4103/0253-7613.42303

**Published:** 2008-06

**Authors:** Jude E. Okokon, Ette Ettebong, Bassey S. Antia

**Affiliations:** Department of Pharmacology and Toxicology, Faculty of Pharmacy, University of Uyo, Uyo, Nigeria; 1 Department of Chemistry, University of Uyo, Uyo, Nigeria

**Keywords:** Antimalarial, malaria, *Plasmodium berghei*, *Stachytarpheta cayennensis*

## Abstract

**Objective:**

To evaluate the *in vivo* antiplasmodial activity of the ethanol leaf extract of *Stachytarpheta cayennensis* in the treatment of various ailment in Niger Delta region of Nigeria, in *Plasmodium berghei* infected mice.

**Materials and Methods:**

The ethanolic leaf extract of *Stachytarpheta cayennensis* (90-270 mg/kg/day) was screened for blood schizonticidal activity against chloroquine sensitive *Plasmodium berghei berghei* in mice. The schizonticidal effect during early and established infections was investigated.

**Result:**

*Stachytarpheta cayennensis* (90-270 mg/kg/day) exhibited significant (*P*< 0.05) blood schizonticidal activity both in 4-day early infection test and in established infection with a considerable mean survival time comparable to that of the standard drug, chloroquine, 5 mg/kg/day.

**Conclusion:**

The leaf extract possesses significant (*P*< 0.05) antiplasmodial activity which confirms it's use in folkloric medicine in the treatment of malaria.

## Introduction

*Stachytarpheta cayennensis* (L.C. Rich) Vahl is a weedy (and sometimes perennial) herbaceous plant from the Verbenaceae family commonly called Brazilian tea. Two common very similar species of *Stachytarpheta cayennensis* grow in the tropics and are use interchangeably (and share the same common names)in the herbal medicine systems of many countries, *Stachytarpheta cayennensis* and *Stachytarpheta jamaicensis*. Ethnobotanically, *Stachytarpheta cayennensis* is used to treat various ailments such as inflammation, pain, fever, hepatic and renal disorder, helminthiasis, constipation, hypertension, stress and diabetes.[1–4] The plant is use in parts of southern Nigeria and Peru[5] for the treatment of malaria. Phytochemical studies of the plant revealed that it contains alkaloids,[6] Ipolamide, beta hydroxyipolamide and verbascoside,[7,8] steroids, triterpenes and irridoids.[9] *Stachytarpheta cayennensis* has been reported to be antiinflammtory, antinociceptive, anti ulcerogenic,[8,10,11] antidiarrheoal[12] as well as sedative[13] and hypotensive.[14] An insignificant *in vitro* antiplasmodial activity has been reported of the plant in Peru.[5] The aim of the present study was to evaluate the *in vivo* antiplasmodial potential of *Stachytarpheta cayennensis* considering its wide acceptability as malarial remedy in southern Nigeria.

## Materials and Methods

### Plant material

Fresh leaves of *Stachytarpheta cayennensis* were procured at Uyo main market, Uyo - Akwa Ibom State of Nigeria in June, 2006 and authenticated by Dr Margaret Bassey, a taxonomist in the Department of Botany, University of Uyo, Uyo, Nigeria. A voucher specimen has been deposited in the faculty of Pharmacy hebarium, University of Uyo, Uyo. The plant material was air dried at room temperature and then powdered.

### Preparation of extract

The dried and powdered leaf of *Stachytarpheta cayennensis* (1 kg) was exhaustively macerated in 70% ethanol for 72h. The liqiud extract obtained was concentrated in vaccum at 40°C.The yield was 0.48%. The extract was stored in a refrigerator at 4°C until used for experiment reported in this study. The dry ethanolic extract was dissolved in distilled water to make the stock solution from which the various doses administered were prepared for use by serial dilution.

### Animals

Albino swiss mice (21-28g) of either sex were obtained from the University of Uyo animal house. They were maintained on standard animal pellets and water ad libitum. Permission and approval for animal studies were obtained from the College of Health Sciences Animal Ethics committee, University of Uyo

### Parasite inoculation

The chloroquine - sensitive *Plasmodium berghei berghei* was obtained from National Institute of Medical Research, Lagos, Nigeria and maintained in mice. The inoculum consisted of 5x10^7^ *P. berghei berghei* parasitized erythrocytes per ml. This was prepared by determining both the percentage parasitaemia and the erythrocytes count of the donor mouse and diluting the blood with isotonic saline in proportions indicated by both determinations. Each mouse was inoculated on day 0, intraperitoneally with 0.2 mL of infected blood containing about 1 × 10^7^ *P. berghei beghei* parasitized red blood cells.

### Determination of LD_50_

The LD_50_ of the extract was determined using albino mice by intraperitoneal (i.p.) route using the method of Lorke.[15]

Evaluation of Schizontocidal Activity on early infection (4 - day test)

Schizontocidal activity of the extract was evaluated using the method described by Knight and Peters.[16] Each mouse was inoculated on the first day (day 0), intraperitoneally, with 0.2 ml of infected blood containing about 1x10^7^ *P. berghei berghei* parasitized erythrocytes. The animals were divided into five groups of five mice each and orally administered, shortly after inoculation, with 90, 180 and 270 mg/kg/day doses of the *Stachytarpheta cayennensis* leaf extract, chloroquine 5 mg/kg/day and an equivalent volume of distilled water (negative control) for four consecutive days, (day 0 to day 3). On the fifth day (day 4), thin films were made from the tail blood of each mouse and the parasitaemia level was determined by counting the number of parasitized erythrocytes out of 200 erythrocytes in random fields of the microscope. Average percentage chemosuppression was calculated as $100\left(\frac{\text{A}-\text{B}}{\text{A}}\right)$, where A is the average percentage parasitaemia in the negative control group and B, average percentage parasitaemia in the test group.

### Evaluation of schizontocidal activity in established infection (Curative or Rane test)

Evaluation of curative potential of the extract was done using a method similar to that described by Ryley and Peter.[17] The mice were injected intraperitoneally with standard inoculum of 1x10^7^ *P. berghei berghei* infected erythrocytes on the first day (day 0). Seventy-two hours later, the mice were divided into five groups of five mice each. The groups were orally administered with *Stachytarpheta cayennensis* leaf extract (90,180, 270 mg/kg/day), chloroquine(5 mg/kg) was given to the positive control group and an equal volume of distilled water to the negative control group. The drug/extract was given once daily for 5 days. Thin films stained with Giemsa stain were prepared from the tail blood of each mouse daily for 5 days to monitor the parasitaemia level. The mean survival time for each group was determined arithmetically by finding the average survival time (days) of the mice (post inoculation) in each group over a period of 28 days (day 0 to day 27).

### Statistical analysis

Data obtained from the study were analyzed statistically using one-way ANOVA followed by a post test, Tukey-Kramer multiple comparison test and values of *P*< 0.05 were considered significant.

## Results

### Acute toxicity

The extract (500-1000 mg/kg) produced physical signs of toxicity such as writhing, gasping, palpitation, decreased respiratory rate, body and limb tone and death depending on the dose. All the mice treated with 4000 mg/kg dose of the extract and above died. The i.p LD_50_ of the extract in mice was calculated to be 938.08/kg.

### 4-day test

Ethanolic leaf extract of *Stachytarpheta cayennensis* produced a dose dependent chemosuppressive effect at various doses employed in this study. The chemosuppression were 64.6, 77.42 and 78.2% for 90,180 and 270 mg/kg/day doses. The chemosupression produced by the extract were significant (*P* < 0.05) compared to control and comparable to that of the standard drug (chloroquine 5 mg/kg/day) with a chemosuppression of 87.8% [Table 1].

**Table 1:** Antiplasmodial activity of *Stachytarpheta cayennensis* during 4-day test

*Drug/extract*	*Dose (mg/kg/day)*	*Average (%) parasitaemia*	*Average (%) suppression*
*Stachytarpheta cayennensis* extract	90	15.66±4.02*	64.6
	180	10.0±3.74*	77.42
	270	9.66±4.49*	78.2
Chloroquine (standard)	5	5.39±1.73*	87.8
Distilled water (control)	0.2 ml	44.3±3.08	-

Data are expressed as mean±S.D for five animals per group.F=98.025

* *P*<0.001 when compared to control

### Curative test

On established infection, it was observed that there was a daily increase in parasitaemia of the control group. However, there was a daily reduction in the parasitaemia levels of the extract treated group as well as that of positive control (chloroquine).

**Figure 1:**
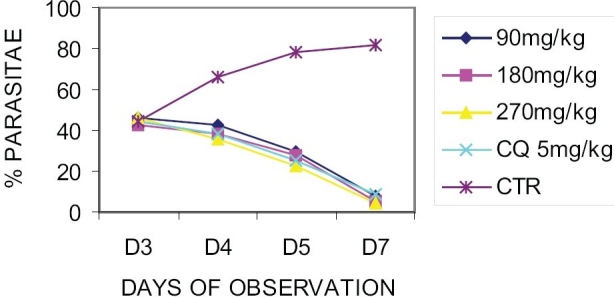
Effect of *Stachytarpheta cayennensis* on established infection (curative test)

On day 7, the average percentage parasitaemia for the groups were 7.6, 5.0, 4.6, 5.0 and 82.0% for 90, 180, 270 mg/kg/day of the extract, chloroquine and control groups respectively [Figure 1]. The mean survival time of the extract treated groups was significantly (*P* < 0.05) longer than that of control and was comparable to that of the standard drug, chloroquine. The values are given in Table 2.

**Table 2:** Mean survival time of mice receiving various doses of ethanolic leaf extract of *Stachytarpheta cayennensis*

*Drug/extract*	*Dose (mg/kg/day)*	*Mean survival time (day)*
*Stachytarpheta cayennensis* extract	90	13.0±2.00
	180	19.0±3.51*
	270	28.0±0.00*
Chloroquine (standard)	5	28.0±0.00*
Distilled water (control)	0.2 ml	9.81±1.32

Data are expressed as mean±S.D for five animals per group.F=97.189

* *P*<0.001 when compared to control

## Discussion

The results show that *Stachytarpheta cayennensis* leaf is moderately toxic as shown in its LD_50_ value of 938.08/kg[18] and also possesses a significant (*P*<0.05) antiplasmodial activity as evident from the chemosuppression obtained during the 4-day early infection test. The leaf extract also exhibited significant curative effect in established infection comparable to the standard drug, chloroquine (5 mg/kg/day) as demonstrated in the mean survival time of the mice in the extract and chloroquine treated groups. *Stachytarpheta cayennensis* leaf has been reported to contain alkaloids,[6] Ipolamide, beta hydroxyipolamide and verbascoside,[7,8] steroids, triterpenes and irridoids.[9] Antiplasmodial screening of plants have implicated alkaloids, terpenes and flavonoids in this activity.[19,20] Although the mechanism of action of this extract has not been elucidated, some plants are known to exert antiplasmodial activity either by causing red blood cell oxidation[21] or by inhibiting protein synthesis[22] depending on their phytochemical constituents. The extract could have exerted its action through either of the two mechanisms mentioned above or by some other unknown mechanism. These compounds may be acting singly or in synergy with one another to exert antiplasmodial activity observed in this study. Thus the active principle needs to be identified.

## Conclusion

The results of this study have shown that the ethanolic leaf extract of *Stachytarpheta cayennensis* possesses antimalarial activity as seen in its ability to suppress *Plasmodium berghei* infection in the two models evaluated. This justifies the traditional usage of this plant as malarial remedy.
